# Riboregulation: a non-canonical tau function

**DOI:** 10.1186/s13024-026-00949-x

**Published:** 2026-06-19

**Authors:** Katherine R. LeBlanc, Randall J. Eck, Sarah J. Benbow, Brian C. Kraemer

**Affiliations:** 1 Molecular & Cellular Biology Graduate Program, University of Washington, Seattle, WA 98195 USA; 2 Medical Scientist Training Program, University of Washington, Seattle, WA 98195 USA; 3 Division of Gerontology and Geriatric Medicine, Department of Medicine, University of Washington, Seattle, WA 98104 USA; 4 Graduate Program in Neuroscience, University of Washington, Seattle, WA 98195 USA; 5 Geriatrics Research Education and Clinical Center, Veterans Affairs Puget Sound Health Care System, Seattle, WA 98108 USA; 6 Department of Laboratory Medicine and Pathology, University of Washington, Seattle, WA 98195 USA; 7 Department of Psychiatry and Behavioral Sciences, University of Washington, Seattle, WA 98195 USA; 8 Department of Genome Sciences, University of Washington, Seattle, WA 98195 USA

**Keywords:** Tauopathy, Alzheimer’s disease, RNA metabolism

## Abstract

Almost since its discovery, tau protein has perplexed scientists and clinicians with its varied roles in physiology as well as its appearance as phosphorylated protein aggregates of various structures in many neurodegenerative diseases. Tau plays a role in microtubule stabilization, but from the earliest of studies, tau has also been observed to bind to RNA, with recent research suggesting tau has a higher affinity for some RNA species compared to microtubules. In the context of disease, tau dysfunction potentiates disruptions to RNA metabolism, including the perturbation of mRNA splicing, impairment of translation, de-repression of transposable elements, and alteration of RNA export and degradation. Tau aggregates directly sequester diverse RNA species and RNA binding proteins. Emerging evidence reinforces the characterization of tau as an RNA binding protein, highlighting questions about both the physiological and disease-related functions of this direct RNA binding. The disparate structure of tau in normal and various disease states makes teasing apart the various impacts on RNA and regulation a more difficult puzzle requiring future study. In this review, we summarize the evidence for tau’s role in RNA biology, including as an RNA binding protein.

## Introduction

### Tau structure and function

The tau (tubulin associated unit) protein was first identified for its role associating with and serving to stabilize microtubules [1]. The *MAPT* gene encodes the tau protein in humans. Tau protein is divided into four domains: the N terminal projection domain, the proline rich domain, the microtubule binding domain, and the C terminal domain. The tau primary RNA transcript undergoes extensive alternative RNA splicing leading to the six predominant isoforms detected in brain neurons. The N terminal projection domain contains two alternatively included regions, N1 and N2, while the MT binding domain contains four repeat sequences and tau isoforms contain three or four repeat sequences (3R and 4R tau) [2]. In the adult brain, 3R and 4R are roughly equal in prevelance [3]. In neurons, tau promotes axonal outgrowth [4].

### Tau pathology in neurodegenerative disease

Detergent insoluble aggregates of tau protein accumulate in a number of different neurodegenerative diseases, collectively termed tauopathies. Tauopathies include primary tauopathies, where tau is the main aggregating protein, like Pick’s disease and progressive supranuclear palsy, and secondary tauopathies, where tau is only one of the aggregating proteins, like Alzheimer’s disease [3]. In these diseases, tau protein aggregates despite normally being unusually soluble even at high concentrations. The makeup, as well as structure, of the aggregates depends on the type of neurodegenerative disease [3]. Tauopathies can be further divided by the predominate isoform of tau present in the disease. For instance, Pick’s disease is both a 3R and primary tauopathy, while Alzheimer’s disease is 3R/4R and secondary tauopathy [3].

There are many features distinguishing tauopathies including the type of tau isoform, activation of astrocytes, presence of neurofibrillary tangles, and the brain region affected, making it difficult to narrow down a single common molecular mechanism driving neuronal dysfunction. Furthermore, tau filaments display various structures in different tauopathies [5]. Cryo-EM structures for tau filaments from sporadic and inherited AD showed a common fold with two C-shaped protofilaments with a combined cross-β/β-helix structure [6]. This was quickly followed by discoveries of novel and unique folds in Pick’s disease [7], chronic traumatic encephalopathy [8], cortical basal degeneration [9], and progressive supranuclear palsy [5]. In Alzheimer’s disease, tau neurofibrillary tangles increase in tandem with neuron loss with respect to duration and severity of disease, while senile plaques show no correlation with disease progression [10]. CA1 neurons with neurofibrillary tangles (NFTs) have altered mRNA expression profiles, especially for proteins involved in AD neuropathology [11], highlighting a cell specific response to tau pathology.

### MAPT mutations in neurodegenerative tauopathies

Findings that mutations in tau can cause familial neurodegenerative disease provide evidence for a causative role of pathological tau in disease. A variety of mutations in *MAPT* coding and noncoding regions (splice sites) have been found in families with autosomal dominant frontotemporal dementia and parkinsonism linked to chromosome 17 (FTDP-17) [12–15]. The observation that tangles were made up of tau was instrumental to uncovering the genetic underpinnings of the familial syndrome.

### Disruptions to RNA metabolism in tauopathy

Beyond high affinity tau binding to RNA, tau dysfunction can contribute to disruptions to key aspects of RNA metabolism, with disruptions to RNA processing driven by tau aggregation thought to contribute to neurodegeneration in disease [16]. For example, tau interacts with critical spliceosome components both in Alzheimer’s disease brains and a *Drosophila* model of mutant V337M tau toxicity [17]. These interactions are linked with aberrant mRNA splicing, including cryptic exon inclusion and intron retention, in both human brain and *Drosophila*, where spliceosome dysfunction is sufficient to drive neurodegeneration [17]. Alternative splicing is also disrupted in a mouse model of human tauopathy (PS19) downstream of RNA binding protein dysregulation [18]. In addition to RNA splicing, both wildtype and mutant P301L tau disrupt alternative polyadenylation, the site selection for poly(A) tail addition, in hnRNP mRNA in an inducible tau expressing HEK cell model [19]. Tau has also been shown to impact m6A-dependent circRNA formation, mRNA translation rates, transposable element–derived dsRNA accumulation, nonsense-mediated mRNA decay, and mRNA export [20–23]. Taken together, these studies suggest tau aggregation disrupts RNA metabolism, RNA processing dysfunction is relevant to disease, and future research is needed to identify the molecular mechanisms – both indirect and possibly direct – by which tau interacts with and regulates RNA.

## Tau Binds to RNA

### Tau RNA binding targets and motifs

From the very earliest studies of the tau protein, clear evidence of RNA interaction with tau has been demonstrated [24]. Indeed, tau/RNA interactions can sequester tau from polymerizing tubulin in vitro and may drive tau into non-productive pathological assemblies [24–26]. Although tau aggregation appears central to tauopathies, the molecular mechanisms driving its seeding and fibrilization have long been a subject of research. One of the earliest lines of evidence suggesting a role for RNA in tau pathology was the finding that RNA can enhance the formation of Alzheimer’s-like pair helical filaments, and is a potent polyanion trigger of aggregation in vitro [27]. More specifically, both 3R and 4R tau form fibrils with parallel, in-register arrangement of β-strands, 4R tau can grow onto 3R tau seeds, and RNA can sustain template-assisted growth, binds to the fibril surface, and can be exchanged for heparin, another co-factor that forms fibrils [28]. Furthermore, tau binding to RNA through non-specific electrostatic interactions was found to inhibit the activity of tau, potentially contributing to a decrease in tau’s normal function [29]. Conversely, neurons in individuals with AD that contain NFTs show decreased levels of a number of mRNA species [11]. Soluble tau seeds from AD brains were found to be sensitive to breakdown by RNAse, implicating RNA as a component in maintaining the structural integrity of tau aggregates [30]. Not only does the addition of numerous types and species of RNA sufficient to induce tau aggregation, but this aggregation was found to lead to increased toxicity in cell culture [31], highlighting that RNA seems to play a role in tau aggregation, seeding, and toxicity. Although many of the high affinity interactions between tau and RNA are sequence nonspecific, there has long been interest in tau binding to poly(A) mRNA and DNA in the context of microtubule assembly. Microtubules themselves cannot bind poly(A) mRNA but bind with a high affinity to tau. Incubating microtubules with poly(A) RNA was found to remove a protein necessary for microtubule assembly, that was later identified as tau itself [24]. This result was also seen in experiments that added RNA homopolymers and also observed a decrease in microtubule assembly [24], indicating that other RNA species beyond poly(A) are able to bind tau. Further experiments indicated that poly(A) RNA inhibits tau-mediated MT assembly in a dose dependent manner [25]. A variety of RNA species including poly(A), poly(U) and total RNA can trigger tau aggregation and seed tau fibrilization in vitro [30, 32, 33]. The poly(A) triggered seeds and fibrils are sensitive to RNAse and human RNA (as opposed to RNA from other sources or heparin) produced more stable tau conformations [30]. Tau and RNA have high affinity binding interactions and tau prefers single stranded RNAs like poly(A) or poly(U), over more structured RNAs like tRNA (summarized in Table 1) [25]. Tau aggregates derived from human cells or transgenic P301L tau mouse brains were enriched for small nuclear RNA species (including snoRNA and snRNA) as well as RNA derived from Alu transposable elements [26]. Experiments over the past 50 years have confirmed that tau binds readily to RNA, however, significant investigation into other RNA species and their effects on toxic tau has yet to be done.

**Table 1:** Documented Tau/RNA interactions

RNAs bound by tau	References
Small nuclear RNA	[26, 34, 35]
PolyA RNA	[25, 30]
Transfer RNA	[34]
Transposon RNA	[26]
Ribosomal RNA	[34, 36]
m6A RNAs (via HNRNPA2B1)	[37]

In addition to poly(A) binding, evidence from a transcriptome wide analysis of tau-RNA binding via cross-linked immunoprecipitation experiments indicated that P301L mutant tau bound to many abundant RNA species including tRNA, specifically tRNA-Arg, with a preference for anti-codon regions in HEK cells and hiPSC neurons [34]. However, these results still indicated that tau likely binds many other forms of RNA, confirming general sequence non-specific interactions identified in various other studies, but suggesting that tau may bind with more specificity to certain RNA species. Further research to identify enriched tau RNA binding motifs, if any such exist, is required. Isolation of tau aggregates from human cells or transgenic P301L tau mice brains followed by RNA sequencing has suggested tau aggregates are enriched for some RNA species including snoRNA and snRNA as well as some transposable elements [26]. Aggregates of the snRNA-specific 2,2,7-trimethylguanosine cap have been observed to co-localize with tau and paired helical filaments in Alzheimer’s disease brains, substantiating sequencing evidence of small regulatory nuclear RNAs mis-localizing to cytoplasmic tau aggregates [35]. Another study investigating more functional outcomes of tau-RNA binding found that tau can sequester ribosomal components, decrease the physical integrity of the ribosome, and lead to tau-rRNA-ribosomal protein aggregates [36]. Although tau binds to rRNA, addition of heparin or tRNA decreased the effect on the ribosome, indicating that other RNA species or polyanions, can outcompete rRNA for tau binding [36], lending even more evidence for the importance of electrostatic interactions in tau-RNA binding.

### Tau RNA binding domains

Early investigation into tau fibrilization indicated that tau can form PHFs with only a truncated version consisting of three repeats [27]. Addition of polyanions, like RNA, decreased the time it took to form PHFs and also induced formation in 3R and 4R tau, with and without the N and C-terminal domains, overcoming previous failure to create PHFs with some of these tau species [27]. The number of tau repeats determined aggregation, even in the presence of RNA. Three repeats led to PHF assembly, while two decreased assembly, and constructs with only one or no repeats did not form filaments [27]. These were the first experiments to demonstrate that the repeat binding domains are necessary and sufficient for PHF aggregation in the context of RNA.

**Fig. 1:**
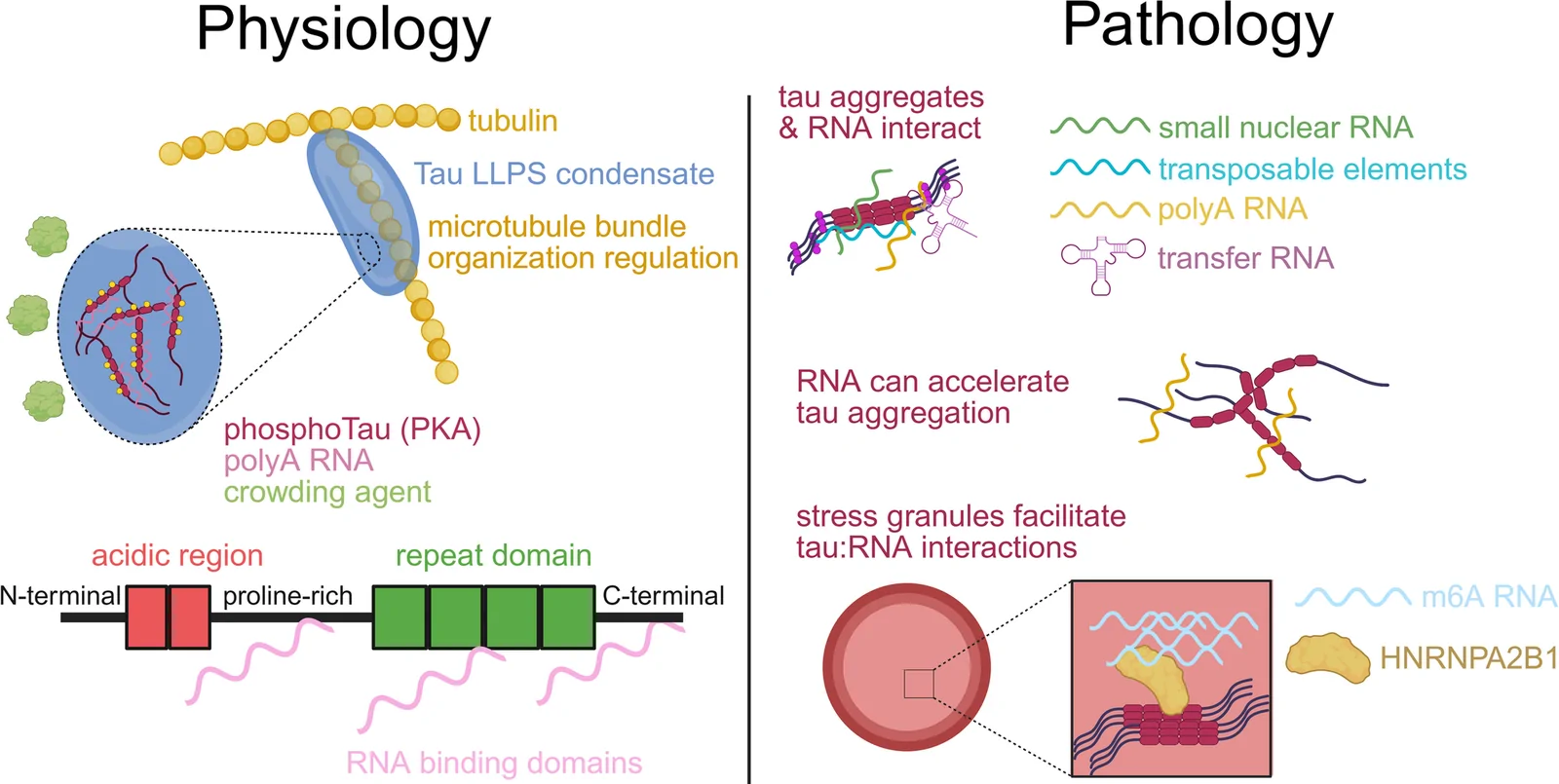
Physiological and Pathological Associations of tau with RNA. Created with BioRender.com

In 2006, Wang et al. conducted a study to attempt to understand tau and RNA interactions by systematically inspecting truncated tau species using gel shift assays [29]. These studies indicated that tau constructs containing the proline rich and microtubule binding domains would associate with RNA (through electrostatic interactions) and that lack of these domains would decrease interactions (though not completely abolish them) [29]. These earlier studies attempted to understand required tau binding domains through systematic creation of smaller tau constructs; however, it appears that multiple tau regions may be capable of binding to RNA (Fig. 1), making isolation of specific binding sites more difficult.

Advances in cryo-EM have allowed us to isolate and visualize protein interactions in stark detail. Monomeric full length tau aggregates into amyloid fibrils in the presence of undigested RNA. These fibrils are capable of seeding further tau aggregation in vitro, while RNA on its own does not seed aggregates. Polymerized RNA is an essential component of self-aggregating and seeding capable tau fibrils and tau-RNA fibrils break down with increasing concentrations of RNAse, indicating that RNA is acting as a sort of “molecular glue” [33]. This RNA is part of the final fibril structure and is predicted to bind to a 36-residue, C-terminal fibril core that was previously characterized as a disordered fuzzy coat in previously determined structures of tau [33]. Shorter tau constructs lacking the C-terminal domain show RNA also binds other regions of tau, however leading to fewer fibrils that take longer to develop, and cryo-EM structural analysis suggests the C-terminal region contains the preferred RNA binding domain. Although we have been able to gain more insight into preferred binding domains for RNA on tau, it has become clearer that tau contains several RNA binding sites that may participate in a host of electrostatic mediated sequence non-specific interactions with various species of RNA.

### Function of RNA binding in physiology

Tau can phase separate into liquid-like condensates under physiological ion concentration conditions only in the presence of additional crowding agents [38]. Liquid-liquid phase separation (LLPS) is a process of de-mixing driven by protein-protein and protein-RNA interactions that results in the formation of a distinct, membraneless cellular compartment, such as a nucleolus or nuclear speckle, which are reminiscent of a drop of oil in water [39].

RNA-tau interactions can promote tau LLPS in vitro, but RNA-driven LLPS is sensitive to increasing ion concentrations, including physiological conditions, in the absence of a crowding agent [38, 40]. In vitro, both poly(A) and poly(U) RNA can induce tau LLPS [34]. In human-induced pluripotent stem cell-derived neurons, tau has been shown to preferentially interact with transfer RNAs (tRNA), offering a potential physiologically relevant RNA partner for LLPS. tRNAs can in fact promote tau LLPS into reversible phase separated condensates, much like synthetic polymeric RNA species in vitro [34]. Research suggest that tau’s proline-rich domain participates in LLPS [41]. The proline domain exhibits high arginine and lysine content, which can facilitate electrostatic interactions with RNA and drive phase separation, basic regions occur throughout the tau protein suggesting other regions may contribute to tau RNA-induced LLPS as well, consistent with evidence for RNA binding via alternative tau domains [42]. Tau-tau and electrostatic interactions are also critical to tau LLPS [43, 44]. Once phase separated, tau condensates can accumulate and concentrate microtubules, acting as hubs for microtubule nucleation and bundle formation in neurons, contributing to tubulin stability and plasticity [38, 45]. Future research is required to fully define the physiological conditions and functions of RNA-induced tau LLPS and develop refined tools to detect tau LLPS in vivo. Whether or not tau LLPS is on pathway to tau fibrilization in neurons also remains an open question.

### Regulation of tau RNA binding

Several post-translational modifications regulate RNA-induced tau LLPS in vitro including acetylation, ubiquitination, and phosphorylation. Site-specific mono-acetylation of the four-repeat region of tau at K369 inhibits heparin or RNA-induced tau LLPS in vitro. Synthetically generated mono-acetylation at K353 and K317, but not K311, helps maintain the solubility of long-lived RNA-induced tau condensates [46]. Enzymatic acetylation of the tau repeat domain by lysine acetyltransferase p300 accelerates the dissolution of RNA-induced tau condensates in vitro, preserving tau solubility [42]. Synthetically generated site-specific mono-ubiquitination of the four-repeat region of tau at L311 or L353 also increases the solubility of RNA-induced tau condensates when compared to non-ubiquitinated tau in vitro [47]. Site-specific synthetic-phosphorylation of the four-repeat region of tau at S352 similarly suppresses RNA-induced tau LLPS, while synthetic phosphorylation of S356, but not Y310, increases the solubility of long-lived RNA-induced tau condensates in vitro [46]. Full-length tau enzymatically phosphorylated by CDK5 and PKA, but not GSK3β, shows diminished RNA-induced LLPS compared to non-phosphorylated tau in vitro as well [38]. When a crowding agent is present, enzymatically phosphorylated tau no longer inhibits LLPS, but highly-phosphorylated tau expressed in insect cells still suppresses LLPS in vitro [38]. Phosphorylated tau by mouse brain extract also has a reduced propensity for RNA-indued LLPS, further suggesting phosphorylation can regulate RNA-induced tau LLPS [40] in vitro. Disease-relevant post-translational modifications also regulate RNA-induced tau LLPS, potentially contributing to pathological aggregation in patients, including N-Glycosylation and phosphorylation at S305 [46, 48]. Additional experiments, especially those under physiological conditions in vivo are required to determine how post-translational modifications of tau regulate the affinity and specificity of tau RNA binding in relation to tau-driven LLPS and tau-RNA binding generally. Dysregulation of tau phase separation can drive the transition of tau from liquid-like to solid aggregate. This pathological LLPS – also influenced by tau-RNA interactions – can result in seed-competent and neurotoxic aggregates and is thought to contribute to the early pathogenesis of neurodegenerative disease [49–52]. A recent study found that tau interactions in vitro with the yeast Hsp40 homolog Ydj1 can maintain tau-Ydj1 liquid-like phase separation, even in the presence of poly(U) RNA, which alone promotes a solid-state transition in vitro [53].

### RNA and tubulin competition for tau

The early work by Bryan et al. in 1975 determined that the presence of RNA, functioning as a polyanion, could inhibit microtubule assembly in a manner indicative of a stoichiometric interaction versus an enzymatic interaction [24]. The fact that tubulin and RNA are both polyanionic pointed to an intermediate factor evidenced in this work as a cationic protein that could be removed using a cationic exchanger such as phosphocellulose or carboxymethylcellulose [24]. The increasing dose dependency of inhibition and rescue of inhibition by the addition of tau protein suggested that tau could be the intermediate protein and RNA could compete with tubulin for this limited protein. This hypothesis was tested directly where purified brain tubulin and purified recombinant tau protein were used to assemble microtubules in vitro in the presence of increasing amounts of purified poly(A) RNA [25]. These experiments demonstrated that as the concentration of poly(A) RNA increased, assembled microtubule mass decreased. Surface Plasmon Resonance (SPR) experiments showed that not only can tau directly bind linear homopolymeric RNA and does so with approximately 5-fold greater affinity relative to tubulin whereas tubulin does not show detectable binding to poly(A) RNA. Taken together, these experiments have shown that tau binds RNA and that RNA can effectively compete with tubulin for tau binding and inhibit tau-microtubule interactions including microtubule assembly [25].

## Tau-RNA in disease

### RNA is found in tau aggregates across diverse pathologies

Over 20 years ago, RNA was first identified as a component of Alzheimer’s disease neurofibrillary tangles and senile plaques using staining of postmortem tissue samples [54]. RNA was subsequently identified in pathological inclusions from other tauopathies including Pick bodies (Fig. 1), but were not found in Lewy bodies, Hirano bodies, and cytoplasmic glial inclusions, pointing to a potential disease specific role for RNA in tau pathology [55]. Tau has also been found to colocalize with RNA in cultured human cells [26], again suggesting that there are opportunities for RNA and tau to interact in disease states.

### Tau RNA interactions in disease pathogenesis

Several studies have highlighted a role for tau RNA interactions in the pathogenesis of tau aggregation and disease progression (Fig. 1). For example, tau 2N4R binding of poly(A) homopolymers in vitro is sufficient to increase tau seeding capability in tau P301S biosensor cells. Long term poly(A) tau incubation can result in soluble tau assemblies, whose seeding activity is dependent on RNA stabilization, as it is ablated by RNAse treatment [30]. In addition to stabilizing fibrils, RNA has been shown to sustain fibril growth during elongation in vitro, suggesting RNA is critical to tau monomer recruitment [28]. In vivo, the co-expression of poly(A)_45_ and tau in the neurons of the nematode *C. elegans* exacerbates tau-driven behavioral and neurodegenerative phenotypes by increasing pathological tau accumulation [25]. Knockout of MSUT2, a protein believed to alter poly(A) RNA tail lengths, increases immunoreactivity with an antibody raised against tau/RNA complexes, giving evidence that poly(A) may modulate tau toxicity by driving a conformational change [25]. Staining for tau-RNA complexes in the brains of AD patients found they are insoluble and are found in pathological lesions [25]. Taken together, this research suggests that the occurrence of tau-RNA interactions facilitates tau aggregation and toxicity in disease. Tau may come into contact with RNA in the context of disease through interactions with several ribonucleoprotein (RNP) granules, including stress granules and nuclear speckles, also contributing to RNA metabolism dysfunction.

### Scaffolded interactions with other RBPs

Tau has an extensive protein interactome [56] that may be altered by mutations in various regions [57]; however, increasing evidence suggests that interactions between tau and RNA binding proteins (RBP) may play a role in the pathogenesis of tauopathies. Proteomic studies and investigations of the tau interactome reveal that many RBPs have altered tau interactions during disease [58] and form insoluble aggregates in later pathological stages [59]. Further immunohistochemistry indicated that RBPs colocalize with phospho-tau but not larger tau inclusions [59]. Insoluble RBPs accumulated together with tau aggregates in AD, especially proteins involved in spliceosome assembly, nuclear speckles, and RNA splicing [60]. RBP co-localization with stress granules increases with oligomeric tau seeding [61], indicating that RBP presence might indicate a difference in tau pathology. Chaperone-mediated ER protein homeostasis and impaired autophagy leading to granulovacuolar degeneration is linked to both pTau and RBP accumulation [62]. It seems clear that RBPs directly interact with tau and that these interactions may drive tau toxicity.

A variety of the RBPs have been implicated in tauopathies. One class of RBPs, Musashi proteins, form oligomers in brain tissue and co-localize with tau in disease [63], altering the nuclear transport of Musashi proteins, forming nuclear aggregates, and changing LaminB1, leading to nuclear instability [64, 65]. A number of other RBPs found in nuclear speckles are required for tau toxicity including MSUT2 [66–69], involved in poly(A) RNA metabolism, ALYREF [70], involved in nuclear RNA export, TXNL4A and PRPF-8 [71], core spliceosome factors, and NIPP1 [72], involved in nuclear inhibition of protein phosphatase 1. Knockout of ELAVL4/HuD, known to regulate the translation and expression of numerous genes involved in AD, exacerbates tau pathology in neuronal culture [73]. Many RBPs with basic-acidic dipeptide domains are found in insoluble fractions of AD brains, including U1 small nuclear ribonucleoprotein 70 kDa, which selectively interacts with tau from AD brains [57]. In rapidly progressive AD, RBP SFPQ and phospho-tau displayed extranuclear co-localization [74]. Conversely, interactions between G3BP2, a component in stress granules, and tau are found early in disease and independent of NFT formation [75]. In cultured cells, tau binding with G3BP2 occurs when MT occupancy by tau is low; conversely, pathological tau species accumulate when G3BP2 is lost [75], indicating that this RBP may serve a protective function in tauopathy.

Transactive response DNA-binding protein (TDP-43) has emerged in recent years as another hallmark of neurodegenerative disorders. Mis-localized TDP-43 occurs in the majority of ALS cases and about half of FTLD and AD cases [76–78]. The presence of pathological TDP-43 deposits is found in up to 70% of AD cases in some cohorts [79, 80], and co-occurs with tau prior to clinical symptoms [81], making it a contributing co-pathology and highlighting the potential for interactions between tau and canonical RNA binding proteins. In the hippocampus, TDP-43 pathology frequently occurs in adjacency with phosphorylated tau in NFTs and pretangles [82], TDP-43 pathology can sometime be found in more advanced Braak stages [83], and TDP-43 contributes to volume/uptake mismatch of tau radioligand tracing [84]. Cleaved TDP-43 clearance decreases when microtubule-associated tau is present [85] and tau oligomers may drive TDP-43 cytoplasmic accumulation [86], suggesting a functional connection between the two proteins. Individuals resistant to AD neuropathologic change demonstrate less limbic-predominant age-related TDP-43 encephalopathy neuropathologic change and a *C. elegans* model of TDP-43 and tau co-pathology demonstrated synergistic toxicity leading to more severe neurodegeneration [87, 88].

Interactions with RNA binding proteins may alter tau isoform expression. Expression of two proteins, G3BP1 and IMP1, leads to the formation of ribonucleoprotein (RNP) granules, concentrating multivalent proteins and RNA. The formation of these granules alters tau isoform expression, increasing the ratio of high molecular weight to low molecular weight tau protein [89]. The presence of RNP granules has functional impacts: it is associated with increased neurite formation and enhanced process growth, highlighting another potential function of tau in health and disease.

### Disruption of stress granules in tauopathy

One site of tau-RNA interactions in cells is within ribonucleoprotein (RNP) granules, including stress granules (SGs). SGs dynamically sequester RNAs and proteins into phase-separated cellular compartments in response to oxidative or other stress or when translation initiation is inhibited, promoting cellular fitness [90, 91]. Several studies have demonstrated co-localization of tau with TIA1-positive SGs [92–94]. Previous work suggests hyperphosphorylated tau is more likely to associate with SGs and that tau internalization delays SGs clearance [94]. In vitro tau interactions with TIA1 can also promote tau LLPS in the absence of crowding agents, and in the presence of RNA, which are abundant in in vivo SGs, the formation of toxic oligomeric tau [95]. In addition to serving as sites of pathological tau LLPS, SGs may also facilitate the toxicity of oligomeric tau by promoting interactions with N6-methyladenosine (m6A) RNA through a scaffolding RBP, HNRNPA2B1, further promoting a translational stress response. Evidence for these interactions are present in human post-mortem tissue in addition to animal and cellular models [37]. Knock-down of HNRNPA2B1 in primary cortical neurons expressing tau with a light-inducible aggregation domain and in the hippocampus of PS19 mice injected with oligomeric tau resulted in less association between tau and m6A as well as pathology including reduced cleaved caspase 3, indicative of apoptosis or neuron death, further suggesting a role for tau RNA interactions in disease [37].

### Role of m6A in tau-mediated disruptions to RNA metabolism

N^6^-methyladenosine (m6A) is a post-transcriptional regulatory modification found on many different RNA species. It is abundant in the brain and plays a role in a number of broad processes, including splicing and stability, as well as more specific roles in neuronal development and neurodegenerative disease [96, 97]. There are a number of methyltransferases that methylate adenine residues including METTL3, METTL14, WTAP24, KIAA1429, METTL16, RBM15, and ZC3H13 and methyl groups are removed by demethylases including FTO and ALKBH5 [97]. Investigations in mouse models of AD indicate that APP/PS1 animals have elevated m6A methylation in the cortex and hippocampus [96] as well as altered m6A methylation of circularRNAs, especially in pathways that could be related to the pathogenesis of AD [98]. A mouse model of beta amyloid and tau co-pathology displayed increased m6A RNA methylation, mainly in the neuronal soma, at six months of age [99]. In the cingulate cortex of elderly mice and individuals with AD, decreased m6A RNA methylation was observed, especially in genes related to synaptic function and reduced m6A levels themselves inhibited synaptic function [100], highlighting that m6A may be altered differentially across brain regions. A large GWAS study identified 258 m6A-SNPs that overlapped with six differentially expressed genes from AD datasets [101], reinforcing the potentially heterogenous impacts of m6A alteration on AD gene expression and pathology.

m6A levels may be driven by altered levels of methyltransferases, demethylases, or other interactor proteins. METTL3 RNA and protein levels are significantly decreased in the hippocampus of AD postmortem brains and METTL3 is found in AD-derived insoluble fractions, correlating with levels of insoluble tau [102, 103]. APP/PS1 mice exhibit increased expression of METTL3 and decreased expression of FTO [96]. Mice with tau and beta amyloid co-pathology have increased METTL3 and decreased ALKBH5 [99]. IGF2BP2, an m6A reader, displays increased expression in AD patients [104]. A large study investigating 25 common m6A RNA methylation regulators identified two clusters of patients with different m6A regulator patterns and these two groups had different immune profiles [105], highlighting that m6A RNA methylation may help define AD subtypes.

m6A has been observed to have contrasting effects. Nasu-Hakola disease, caused by mutations in TYRO protein kinase-binding protein (TYROBP) is a genetic disorder similar to AD with cognitive deficits. *Tyrobp–/–* mice demonstrate learning and memory deficits, elevated levels of tau/phosphorylated tau and amyloid beta, and decreased m6A methylation, probably via decreased expression of METTL3, METTL14, and WTAP [106]. Loss of METTL3 in macrophages improved cognitive function in APP/PS1 mouse model of amyloid beta plaque formation [107]. In cells treated with amyloid beta and APP/PS1 mice, METTL3 upregulation via KMDA1 increased phosphorylated tau clearance via autophagy [108]. In a mouse model of AD, METTL3 knockdown led to memory deficits, synaptic loss, and neuronal death. Inhibition of oxidative stress and the cell cycle decreased neuronal damage in primary neurons exposed to amyloid beta, as did inhibition of m6A demethylation. METTL3 overexpression prevented synaptic damage and cognitive impairment in a mouse model using amyloid beta addition [103].

### Role of TIA1 in tau RNA binding

Tau is present in stress granules, and specifically interacts with RNA binding protein T-cell intracellular antigen 1 (TIA1) [93, 109]. TIA1 functions in the innate immune system during cellular stress, but significantly less is known about its role in neuroinflammation. A functional network analysis suggested that TIA1 requires tau for normal interactions with RNA metabolism machinery [93]. Addition of tau accelerates stress granule formation while TIA1 knockdown inhibits tau misfolding and toxicity in cell culture [93] and in P301S Tau mice [109]. TIA1 overexpression stimulates tau misfolding, inclusion of phospho-tau, and neurodegeneration [93, 110]. Furthermore, pharmacological prevention of stress granule formation prevents tau pathology [93]. The reduction of TIA1 lead to decreased size and number of stress granules [109]. TIA1 seems to be crucial in the production of tau oligomers and the neuronal response to toxic tau in vivo [111]. PS301S transgenic mice with knockdown or knockout of TIA1 display increased neuroinflammation in early and advanced disease states [112], indicating that TIA1 may play a role in mediating the inflammatory response to tau. In tau transgenic mice, as well as human AD and FTLD-tau, stress granules with TIA1 colocalize with tau pathology during later stages of the disease. In contrast, stress granules with G3BP increase in neurons as the disease progresses but never colocalizes with tau pathology [110].

### Disruption of nuclear speckles in tauopathy

In addition to stress granules, nuclear speckles (NS) are another RNP granule disrupted in human tauopathy disorders [26, 113]. Nuclear speckles are phase-separated, non-membrane bound nuclear organelles responsible for the regulation of gene expression and RNA processing, including RNA splicing and export [114]. NS are also hubs of proteostasis, concentrating ubiquitin proteasome system (UPS) components, and coordinating proteostatic gene expression [115, 116]. The RNA binding proteins SON and SRRM2 form the NS scaffold and are required for NS formation [117, 118]. In HEK293 cells transfected with brain homogenates from PS19 mice expressing aggregate-prone human mutant tau (P301S), SRRM2 mislocalizes from NS to tau aggregates disrupting NS dynamics and mRNA splicing [26]. Several other disease-relevant NS components also mislocalize from NS to tau aggregates in this model including PNN, SFPQ, MSUT2, and DYRK1A, likely contributing to NS dysfunction [26]. In addition to containing NS components, tau aggregates in this HEK293 biosensor model and tau mutant P301L mouse brains are enriched for NS-associated small non-coding RNAs (snRNA) [26]. While it was initially thought SRRM2 interacted with tau aggregates first in the nucleus, recent research suggests the mislocalization of SRRM2 to the cytoplasm, driven by inflammation or other stress, precedes tau co-localization [113, 119, 120]. Once mislocalized, SRRM2 can in fact seed tau aggregation via its polyserine repeat domains in HEK293 tau biosensor cells and facilitate tau interactions with snRNAs [119]. Several other studies also find snRNAs associated with tau or neurofibrillary tangles in human cells or Alzheimer’s disease brains [34, 35]. In Alzheimer’s disease patients, the mislocalization of SRRM2 in the frontal cortex is associated with increased tau pathology and an earlier age of disease onset, suggesting disruptions to NS drive disease progression and restoration of NS homeostasis could be protective against tauopathy [26, 113, 121]. Further investigation into tau snRNA interactions seems warranted.

## Conclusion

### Tau RNA binding competes with MT binding

#### Ribostasis

In non-disease states, tau-RNA interactions may play a role in regulating tau-MT binding; an association that is known to be highly regulated through several mechanisms including tau splice isoform ratios and posttranslational modifications. The higher affinity tau-RNA interaction would provide a highly effective tool to sequester tau with distinct utility in the regulation of MT dynamics. RNA binding of tau may prevent excess tau-MT binding and help reduce tau-induced MT growth and mass accumulation when a more stable microtubule population is needed.

#### Ribopathology

Several lines of evidence have shown that RNA plays a role in the dysregulation and pathological activity of tau. RNA can induce tau aggregation and increase toxicity, but the precise mechanisms underlying RNA’s role in neurodegenerative pathology remains incompletely understood. The normal balance of competition between RNA and MTs for tau appears upset by pathological events occurring in disease, potentially leading to prolonged RNA-tau exposure. In addition, increased affinity for RNA over microtubules may perpetuate tau loss of function in disease contexts (Fig. 1). In turn the resultant tau-RNA assemblies lead to formation of toxic tau conformations including fibrils and the over-sequestration of tau leading to a loss of MT related functions. Either of these contexts may be detrimental to the regulation of normal neuronal physiology. Conversely, tau pathology drives RNA mis-splicing, highlighting potential for amplification of ribopathology (Fig. 1). Further molecular dissection of the neurodegenerative pathways resulting from loss of ribostasis will be critical to development of new therapeutic ideas for tauopathy.

### RNA binding proteins and pathological tau

Tau interacts with many distinct RBPs in tauopathies, even forming insoluble aggregates with RBPs during later stage disease. Musashi proteins colocalize with tau in disease and alter nuclear transport, eventually causing nuclear instability. Similarly, tau directly binds to RNA binding protein TIA1, normally found in stress granules, where its interaction is needed for RNA-related mechanisms (Fig. 1). Pathological accumulation of tau drives stress granule formation via TIA1, leading to neurodegeneration. Additionally, a number of RNA binding proteins normally found in nuclear speckles, including SRRM2, PNN, SFPQ, MSUT2, and DYRK1A, mislocalize to tau aggregates in disease. These aggregates also contain RNA species like snRNAs. SRRM2 specifically, once mislocalized, can seed further tau aggregation, acting as a mechanism to drive the progression of tau pathology. The RBP TDP-43 occurs as a common co-pathology in Alzheimer’s disease and recent experiments suggest there may be synergistic toxicity between the two proteins [87, 88], indicating that there may be even more intricate interactions between tau and RBPs still undiscovered.

Increasing evidence also suggests that tau’s myriad interactions with RNA binding proteins may represent mechanisms of the toxic effect of tau in vivo, since many aspects of neurodegeneration can be prevented by altering RNA binding proteins. Knockdown of MSUT2, a well-known RBP, in mouse models of tau decreases memory and learning deficits and prevents neuron loss [69]; MSUT2 has further been shown to modulate tau seeding, indicating the wide ranging effects that RBPs may be having on tau toxicity [122]. Knockdown or mutation of many other RBPs has also been demonstrated to decrease or prevent tau toxicity including ALYREF, NIPP1, TXNL4A, and PRPF8 [70–72].

### Tau-RNA binding as a potential target for neurotherapeutics

Tau has a distributed non-canonical RNA binding domain that contact various mRNAs through sequence non-specific electrostatic interactions. Action of tau as a RBP, as well as its interactions with other RBPs, leads to aggregates of tau, mRNA, and RBPs and decreased variety of mRNA species in neurons that have NFTs. Several problems arise when considering tau-RNA binding as a target for neurotherapeutics. The lack of sequence specificity non-canonical distributed RNA binding domain poses clear barriers to development of tau-RNA binding inhibitors. It also remains unclear whether RNA binding and MT binding are separable activities. Further, it remains uncertain whether tau-RNA binding has a normal biological function that could lead to toxicity if inhibited or if mRNA binding to tau alters the ability of tau to perform its normal physiological role, as RNA-driven LLPS of tau in physiological-like conditions can alter microtubular dynamics. Despite these challenges, there seems a more direct precedent for targeting specific RBPs. Screens for inhibitors of the RNA binding proteins MSUT2 and TIA1 have both shown some promise for early stage drug development in this area [68, 93, 123]. Similar drug screens or drug design can be undertaken for the various other RNA binding proteins that are known to directly modulate tau toxicity.

## Data Availability

No datasets were generated or analysed during the current study.
